# Palliative Pericardiectomy for Constrictive Pericarditis Caused by Squamous Cell Carcinoma of the Breast: A Case Report

**DOI:** 10.7759/cureus.95101

**Published:** 2025-10-21

**Authors:** Shota Inoue, Atsuhiko Sato, Shigefumi Matsuyama

**Affiliations:** 1 Department of Cardiovascular Surgery, Toranomon Hospital, Tokyo, JPN

**Keywords:** breast cancer metastasis, cancer cachexia, case report, heart failure, malignant constrictive pericarditis, pericardiectomy, squamous cell carcinoma (scc)

## Abstract

Constrictive pericarditis caused by malignancy is a rare but serious condition, with limited reports on its palliative surgical management. In particular, pericardial metastasis from squamous cell carcinoma of the breast is extremely uncommon, and the role of pericardiectomy in such cases remains unclear. A 57-year-old woman underwent a left mastectomy for squamous cell carcinoma of the breast at the age of 54 years. Two years later, the patient developed cardiac tamponade and underwent pericardiocentesis. Cytological examination confirmed malignant pericarditis due to recurrent breast cancer. Although partial remission was achieved with chemotherapy, the patient subsequently developed heart failure symptoms, making it difficult to continue the chemotherapy. Echocardiography and chest computed tomography revealed pericardial thickening, whereas the right heart catheterization showed a dip-and-plateau waveform, leading to a diagnosis of constrictive pericarditis. Given that prolonged survival for several years was expected with continued chemotherapy, pericardiectomy was performed. Postoperative pathological examination confirmed pericardial metastasis of breast cancer. Although the patient experienced temporary symptomatic improvement postoperatively, her condition worsened, leading to death on postoperative day 28. This case suggests that even when palliative pericardiectomy is performed for malignant constrictive pericarditis, the prognosis may remain poor owing to progressive heart failure. In particular, postoperative left ventricular dysfunction and cancer cachexia can significantly affect outcomes, necessitating careful consideration of surgical indications.

## Introduction

Reports on pericardiectomy for malignant constrictive pericarditis are rare, and no established consensus exists regarding surgical indications or perioperative management. In general, pericardiectomy is the standard treatment for symptomatic constrictive pericarditis; however, prognosis varies depending on the underlying etiology [1].

Herein, we report a case of pericardiectomy for malignant constrictive pericarditis caused by squamous cell carcinoma of the breast.

## Case presentation

A 57-year-old woman presented with bilateral lower extremity edema, weight gain, and exertional dyspnea. She had a medical history of hyperthyroidism and a left breast cancer diagnosis at the age of 54 years. The clinical stage at the time of diagnosis was cT2N1M0, and she underwent a left mastectomy and axillary lymph node dissection. Histopathology confirmed pT2N0M0 stage IIA squamous cell carcinoma with triple-negative receptor status, high Ki-67 proliferation index (90%), and positive PD-L1 expression. Adjuvant chemotherapy included four cycles of dose-dense doxorubicin and cyclophosphamide, followed by 12 weekly cycles of paclitaxel. Radiotherapy was not administered.

At the age of 56 years, she developed cardiac tamponade, which was managed by pericardiocentesis. Cytological analysis revealed malignant pericarditis due to recurrence of breast cancer. Echocardiography post-drainage demonstrated an estimated right atrial pressure (RAP) of 8 mmHg and a deceleration time of 313 ms, without evidence of pericardial thickening. Computed tomography (CT) imaging revealed multiple pulmonary nodules and enlarged lymph nodes in the right supraclavicular, mediastinal, and bilateral hilar regions, confirming recurrence. Pembrolizumab in combination with gemcitabine and carboplatin was initiated, achieving partial remission with lymph node reduction. However, the patient subsequently developed worsening edema, weight gain, and exertional dyspnea refractory to diuretic therapy. Owing to declining performance status, chemotherapy was discontinued.

On presentation, she was 164 cm tall, weighed 76.3 kg, and had a blood pressure of 103/62 mmHg and a heart rate of 90 bpm. Cardiac auscultation revealed diminished heart sounds without murmurs. Pitting edema was noted in both legs. She was classified as New York Heart Association Class III.

Laboratory tests revealed elevated levels of liver enzymes (aspartate transaminase: 47 U/L, alanine transaminase: 57 U/L, alkaline phosphatase: 182 U/L, gamma-glutamyl transferase: 98 U/L), C-reactive protein (2.54 mg/dL), and brain natriuretic peptide (310.4 pg/mL). The carcinoembryonic antigen level was elevated at 17.1 ng/mL, whereas bilirubin and creatinine were within normal ranges (Table 1).

**Table 1:** Laboratory findings

Blood analysis	Results	Reference range
White blood cells (×10³/μL)	5.9	3.3–8.6
Red blood cells (×10⁶/μL)	3.79	3.86–4.92
Hemoglobin (g/dL)	11.6	11.6–14.8
Hematocrit (%)	36.9	35.1–44.4
Platelet count (×10⁴/μL)	13.8	15.8–34.8
Albumin (g/dL)	2.7	4.1–5.1
Aspartate aminotransferase (U/L)	47	13–30
Alanine aminotransferase (U/L)	57	7–23
Alkaline phosphatase (U/L)	182	106–322
Gamma-glutamyl transferase (U/L)	98	9–32
Total bilirubin (mg/dL)	1.0	0.4–1.5
Blood urea nitrogen (mg/dL)	13	8–20
Creatinine (mg/dL)	0.49	0.46–0.79
Sodium (mEq/L)	138	138–145
Potassium (mEq/L)	3.8	3.6–4.8
C-reactive protein (mg/dL)	2.54	≤0.14
Brain natriuretic peptide (pg/mL)	310.4	≤18.4
Prothrombin time-international normalized ratio	1.29	0.80–1.20
Activated partial thromboplastin time (sec)	33.0	23–40
D-dimer (μg/mL)	18.0	<1.0
Carcinoembryonic antigen (ng/mL)	17.1	≤5.0

**Figure 1:**
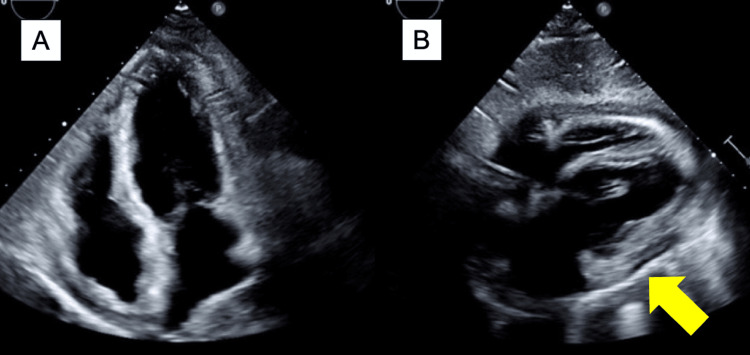
Transthoracic echocardiography (A: apical four-chamber view; B: subcostal four-chamber view) Pericardial thickening (arrow) and diastolic dysfunction were observed. A small pericardial effusion was observed

ECG showed sinus rhythm with low voltage and inverted T-waves in leads II, III, aVf, V5, and V6. Chest X-ray revealed a cardiothoracic ratio of 58% with blunted costophrenic angles. Transthoracic echocardiography (TTE) indicated preserved left ventricular ejection fraction (LVEF) at 59%, left ventricular dimensions within normal limits, mild inferior vena cava (IVC) dilation (21.8 mm) with reduced respiratory variation (6.9%), shortened deceleration time (127 ms), and evidence of pericardial thickening with septal bounce (Figure 1). No significant valvular disease was observed.

**Figure 2:**
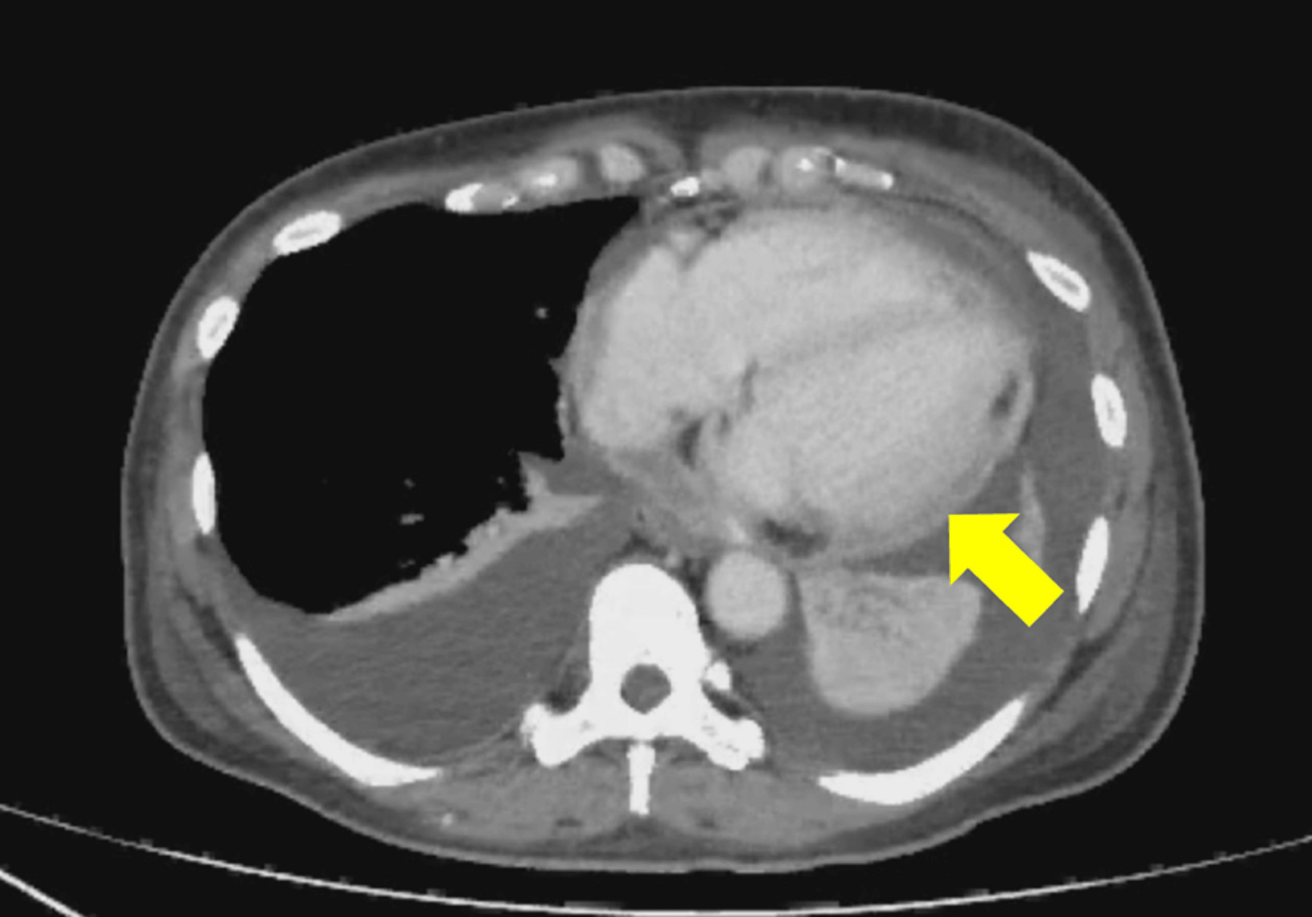
Contrast-enhanced chest and abdominal computed tomography The findings included pericardial thickening (arrow), a small pericardial effusion, bilateral pleural effusions, inferior vena cava dilation, and hepatic congestion.

Contrast-enhanced chest and abdominal CT demonstrated diffuse pericardial thickening with minimal effusion, bilateral pleural effusions, IVC dilation, and hepatic congestion (Figure 2).

Right heart catheterization showed a dip-and-plateau right ventricular pressure waveform, elevated filling pressures (RAP 21 mmHg, right ventricular end-diastolic pressure 22 mmHg, left ventricular end-diastolic pressure 28 mmHg), and a low cardiac index of 1.21 L/min/m².

**Figure 3:**
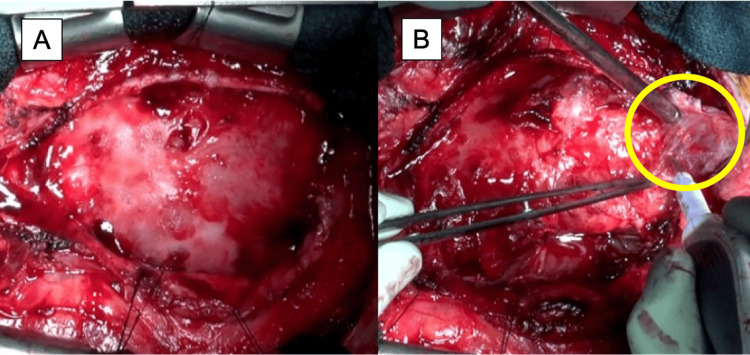
Intraoperative findings Upon opening the pericardium, the whitish lesion was suspected to be a tumor (A). The whitish epicardial lesion (circle) was dissected and excised (B). The myocardium surrounding the whitish lesion was fragile

Although the cancer had shown a partial response to systemic chemotherapy, treatment had to be discontinued owing to the worsening performance status caused by constrictive pericarditis-related heart failure. Given the progressive symptoms of fluid overload and dyspnea, and with the goal of relieving heart failure and potentially resuming chemotherapy, palliative pericardiectomy was planned. The patient was fully informed and expressed a strong desire to undergo the procedure. The pericardium and epicardium over the anterior surface of the right heart showed mild adhesions, which allowed a relatively straightforward dissection (Figure 3). A whitish epicardial lesion suspected of tumor infiltration was excised and submitted for pathological examination. In contrast, dense adhesions were observed around the right ventricular outflow tract, superior vena cava, IVC, left ventricle, and apex. In areas with severe adhesion, a waffle procedure-consisting of grid-like incisions of the epicardium-was performed to relieve constriction. Circumferential pericardial and epicardial resection and incision led to a visible improvement in cardiac expansion, which was confirmed intraoperatively by transesophageal echocardiography showing enhanced diastolic function without a reduction in LVEF. Central venous pressure decreased from 20 mmHg preoperatively to 15 mmHg postoperatively.

**Figure 4:**
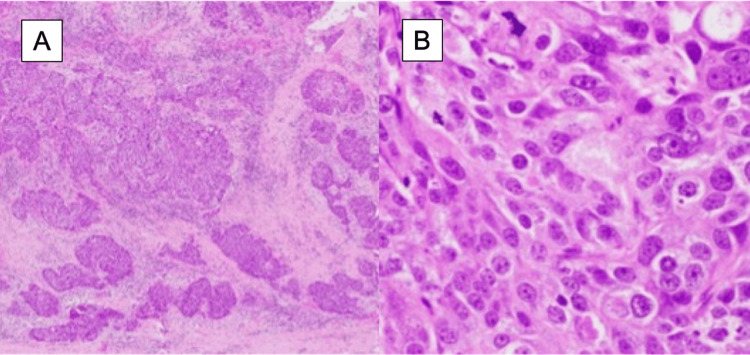
Histopathological findings with hematoxylin and eosin staining (A: low magnification; B: high magnification) Infiltration of squamous cell carcinoma was observed within the pericardial wall, characterized by a solid nest-like proliferation with evidence of coagulative necrosis and occasional single-cell keratinization

Histopathological analysis of the resected pericardium revealed infiltrating non-small cell carcinoma consistent with squamous cell carcinoma, characterized by solid tumor nests with coagulative necrosis and focal keratinization, consistent with recurrent breast cancer (Figure 4).

**Figure 5:**
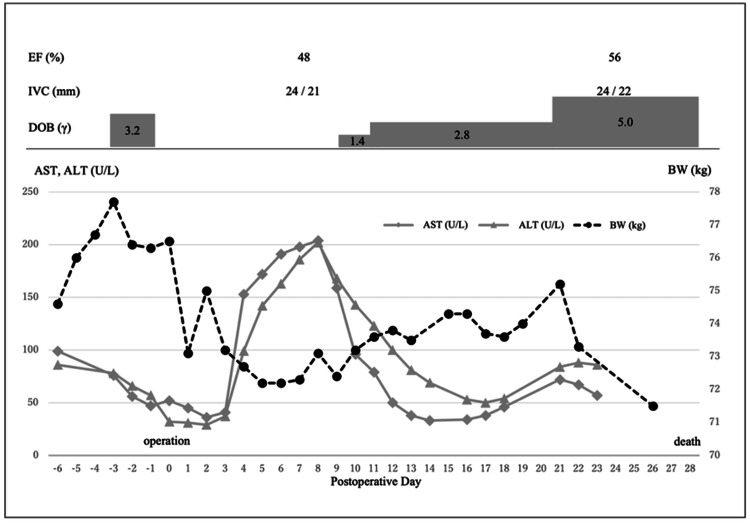
Clinical course Immediately after surgery, body weight and liver enzyme levels significantly decreased. However, as the ejection fraction declined, body weight and liver enzyme levels began to increase, accompanied by an increased demand for dobutamine. EF: ejection fraction; IVC: inferior vena cava; DOB: dobutamine; AST: aspartate transaminase; ALT: alanine transaminase; BW: body weight

Postoperatively, the patient’s dyspnea improved markedly, although peripheral edema persisted. Liver function tests initially improved, and dobutamine support, required preoperatively, was successfully discontinued immediately after surgery. However, by postoperative day four, weight gain and symptoms recurred alongside elevated liver enzymes. TTE at this time showed reduced LVEF (48%). Dobutamine was reintroduced on postoperative day nine. Despite medical management, the patient’s condition progressively worsened, and palliative care was initiated in accordance with her wishes. In consultation with the palliative care team, appropriate opioid therapy was administered to relieve symptoms. The patient remained hospitalized and passed away on postoperative day 28 (Figure 5).

## Discussion

This case study highlights two key points. First, pericardiectomy for malignant constrictive pericarditis may lead to a poor prognosis owing to postoperative left ventricular dysfunction and cancer cachexia. Second, squamous cell carcinoma of the breast is rare, has a poor prognosis, and can also metastasize to the pericardium.

The causes of constrictive pericarditis include idiopathic factors, cardiac surgery, radiation therapy, tuberculosis, chronic renal failure, and malignancies, with malignancy accounting for approximately 4.4% of the cases [1,2]. However, reports of pericardiectomy for malignant constrictive pericarditis are rare. Buyukbayrak et al. reported a case series of eight patients who underwent pericardiectomy for malignant constrictive pericarditis, with lung cancer being the most common primary malignancy [3]. The median postoperative survival was only 14.82 months, with a poor prognosis; three of the eight patients (37.5%) died from heart failure. Furthermore, seven of the eight patients (87.5%) developed postoperative low cardiac output syndrome (LCOS). In contrast, the overall postoperative survival rate for constrictive pericarditis was reported to be 87% at five years and 78% at 10 years [4], with the incidence of LCOS ranging from 22.2% to 28% [2,5]. These findings suggest that malignant constrictive pericarditis carries a particularly high risk of poor postoperative survival and LCOS. LCOS is thought to result from a sudden increase in preload to the left side of the heart owing to a rapid improvement in diastolic function after pericardiectomy [6]. This phenomenon can be explained by the Frank-Starling law [7].

Several prognostic factors for poor survival after pericardiectomy have been reported, including radiation history, renal dysfunction, elevated pulmonary pressures, left ventricular dysfunction, hyponatremia, and advanced age [1,8]. Notably, partial pericardiectomy was associated with a 4.5-fold higher mortality risk compared to total pericardiectomy [8], underscoring the importance of complete resection. In the present case, postoperative echocardiography revealed left ventricular dysfunction. Increased right atrial and ventricular pressures with IVC dilation suggested concomitant right heart failure. These findings, along with elevated liver enzymes and weight gain, indicated biventricular dysfunction. Furthermore, the patient exhibited cancer cachexia preoperatively, and hypoalbuminemia persisted postoperatively. Hypoalbuminemia and tumor-related cytokines [9] likely exacerbated heart failure by promoting fluid leakage into the third space and intravascular dehydration.

According to the World Health Organization classification, squamous cell carcinoma of the breast is classified as a metaplastic carcinoma and is thought to arise from squamous metaplasia of adenocarcinoma [10]. Its incidence is extremely low, accounting for only 0.1% of all breast cancer cases [11]. Compared to invasive ductal carcinoma, squamous cell carcinoma of the breast is characterized by a higher proportion of male patients, greater tumor aggressiveness, larger tumor size, and a higher frequency of lymph node involvement and initial distant metastases [12]. The bone, lungs, liver, and brain are common sites of distant metastasis [12], whereas cardiac metastasis is extremely rare. The presence or absence of distant metastasis is a significant prognostic factor. The five-year survival rate of patients with squamous cell carcinoma of the breast is approximately 40% [13]. However, prognosis is particularly poor in recurrent cases, with reported post-recurrence survival periods ranging from 2 to 86 months (median: 14 months) [13] or 1 to 3.5 years [14]. Noguchi et al. reported that in the longest surviving case (3.5 years post-recurrence), chemotherapy achieved a partial response [14], whereas in other cases, the disease progressed despite chemotherapy. In the present case, chemotherapy for recurrent squamous cell carcinoma of the breast resulted in a partial response, and prolonged survival for several years was anticipated. However, heart failure due to constrictive pericarditis was symptomatic and refractory to medical treatment. Therefore, pericardiectomy was considered an appropriate intervention for symptom relief, depending on the patient's condition and preferences.

Although malignant constrictive pericarditis is occasionally encountered in clinical practice, its surgical indications are limited, and no standardized surgical treatment has been established. Given the potential for poor prognosis owing to postoperative left ventricular dysfunction and cancer cachexia, careful consideration of treatment options is essential. Specifically, if the underlying malignancy is expected to have a relatively favorable prognosis, palliative surgery may be proposed with a thorough assessment of the risk of postoperative heart failure. Although squamous cell carcinomas of the breast are extremely rare, they can metastasize to the pericardium. Therefore, cardiac follow-up is crucial in these cases.

## Conclusions

We report a case of malignant constrictive pericarditis caused by squamous cell carcinoma of the breast, treated with palliative pericardiectomy. Considering the potential for poor postoperative prognosis due to left ventricular dysfunction and cancer cachexia, palliative surgery should be carefully evaluated, with a thorough explanation of the risks and based on the patient’s informed decision.
